# Estimating Variance of Log Standardized Incidence Ratios Assessing Health Care Providers’ Performance: Comparative Analysis Using Bayesian, Bootstrap, and Delta Method Approaches

**DOI:** 10.2196/77415

**Published:** 2025-10-09

**Authors:** Solomon Woldeyohannes, Yomei Jones, Paul Lawton

**Affiliations:** 1 Menzies School of Health Research, Charles Darwin University, Northern Territory, Darwin, Casuarina, 0811, Australia, 61 0424635541; 2 School of Veterinary Sciences, University of Queensland, Gatton, Australia

**Keywords:** standardized incidence ratio, SIR, performance, health care provider, machine learning, equity

## Abstract

**Background:**

In health care providers’ performance assessment, standardized incidence ratios are essential tools used to assess whether observed event rates deviate from expected values. Accurate estimation of variance in these ratios is crucial as it affects decision-making regarding providers’ performance. There is little data on how the choice of these variance estimation methods affects decision-making.

**Objective:**

In this study, we compared 3 methods (the delta method, bootstrapping method, and Bayesian approach) to estimate the variance of the logarithm of the standardized incidence ratio.

**Methods:**

Using patient-level data from the Australia and New Zealand Dialysis and Transplant Registry for 2012‐2023, we used a random effects model to predict treatment at home 1 year after starting treatment. We compared the 3 approaches (with more than 5000 iterations for bootstrapping and Markov chain Monte Carlo sampling) using bias, variance, and mean squared error (MSE) as performance measures. Using the 3 methods, funnel plots were used to compare the hospitals’ performance in treating Indigenous and non-Indigenous patients close to home, as a service-level measure of equity.

**Results:**

The bias values across all methods were similar, with the Bayesian method narrowly having the lowest bias (0.01922), followed by the delta method (0.01927) and bootstrap method (0.02567). In addition, the Bayesian method exhibited the lowest variance (0.00005), indicating more stable and less dispersed estimates. The delta method had a higher variance (0.00016), while the bootstrap method had the highest variance (0.00027), meaning it introduced more uncertainty. Finally, the Bayesian method had the lowest MSE (0.00042), indicating better overall accuracy, while the bootstrap method had the highest MSE (0.00094), showing it was the least reliable method.

**Conclusions:**

We demonstrated that these methods can be used to measure equity for patient-centered outcomes, both within and between service providers simultaneously. The choice of variance estimation method is critical and heavily affects the interpretation of the performance of health service providers. We favor the Bayesian Markov chain Monte Carlo method as it was found to be a better approach.

## Introduction

Public scrutiny of health care service performance has been emphasized in the last two decades. For instance, the Australian government has introduced the National Health Reform in 2011 [1] and recently the 2020‐2025 National Health Reform Agreement [2]. This, in turn, has led to increased attention to institutional comparisons based on quantitative outcome measures such as standardized mortality ratios (SMRs) in which, with the aid of CIs, “outlying” institutions are identified [3]. A league table of hospitals based on mortality [4] and Shewhart’s control charts (using 1, 2, and 3 SD limits) [5] has been proposed and criticized to compare institutional ranking. More recently, a “funnel plot,” in which an estimate of an underlying quantity is plotted against an interpretable measure of its precision, has become a useful graphical aid for institutional comparisons [3,6,7]. Although funnel plots have been used in meta-analyses, in particular to detect publication bias, they have recently been strongly recommended as the most appropriate way to display performance indicators such as comparisons of risk-adjusted rates between health care units [8]. SMRs are the commonly used performance index for institutional comparisons [9]. However, this concept has been readily extended to encompass several other indices such as age-standardized relative survival and excess hazard ratios [8] and standardized incidence ratio (SIR) [10]. Estimating the variance of log SIR (denoted by Log-SIR hereafter) is necessary for creating false discovery rates (FDRs) in studies that use funnel plots for assessing centers’/hospitals’ performance. Accurate estimation of variance in these ratios is crucial as it affects decision-making regarding hospital performance and quality improvement strategies. Despite different variance estimation methods being used widely in application, there are no data on how the choice of these methods affects the assessment of performance. In this study, we compared 3 methods, namely, delta method, bootstrapping, and Bayesian approaches, to estimate the variance of the Log-SIR and subsequent funnel plot approaches to build FDRs for the Log-SIR.

The delta method is the analytical approach to estimate the variance of the logarithm of SMR, denoted by Log-SMR hereafter. It approximates the variance of a function of random variables by using the Jacobian matrix and the covariance matrix of the original variables [11].

Quaresma et al [12] used the delta method directly to estimate risk-adjusted excess hazard ratios as a performance measure in a study of population-based cancer survival. Also, Powell [13] applied the delta method to approximate the variance of demographic parameters in avian biology studies. Vasilevskis et al [9] used CIs for comparing SMR using a bootstrapping approach in a study involving prediction of 30-day intensive care unit mortality. Though CIs can be constructed for SMR directly, Hosmer and Lemeshow [14] demonstrated CIs with good coverage for the logarithm of the SMR. Also, Austin [15] investigated 4 bootstrap procedures for estimating CIs for predicted-to-expected ratios in a hospital profiling study. They indicated that existing bootstrap procedures should not be used to compute CIs for predicted-to-expected ratios when conducting provider profiling.

Like bootstrapping, a Bayesian approach via Markov chain Monte Carlo can be used to approximate this variance. For instance, Ventrucci et al [16] applied Bayesian hierarchical models to estimate small area level SMR and constructed FDRs in a study of liver cancer morbidity cases recorded between 1998 and 2003 in Emilia-Romagna municipalities. In addition, Sukul et al [17] demonstrated the application of a Bayesian hierarchical model in assessing hospital and operator variation in cardiac rehabilitation referral and participation after percutaneous coronary intervention using a retrospective observational cohort of patients who underwent percutaneous coronary intervention at 48 nonfederal Michigan hospitals between January 1, 2012, and March 31, 2018.

Applying the delta method depends on fulfillment of underlying distributional assumption, asymptotic normality. Bootstrapping, on the contrary, has the advantage of not relying on distributional assumptions and can be used to directly estimate the distribution of Log-SIR or Log-SMR. This can lead to more robust variance estimates, particularly in settings with small sample sizes or unknown distributions. By resampling, bootstrapping accounts for sampling variability and can help improve the precision of performance assessments [11]. Therefore, this study compares the 3 variance estimation methods using bias, variance, and mean squared error (MSE) as measures of performance.

## Methods

### Motivating Idea

For more than 25 years, First Nations health organizations and patients in rural and remote Australia have persistently called for more responsive treatment, closer to home, for First Nations people with end-stage kidney disease [18,19]. Community-led advocacy groups have continued this call in more recent years. A national meeting of First Nations patients with kidney failure in September 2017 renewed this message [20]. Over the last 15 years, substantial progress has been made in expanding and decentralizing hemodialysis care across remote Australia [21]. Nevertheless, most treatment is still provided as hemodialysis in nurse-facilitated centers in major or regional towns, rather than at home in remote communities [22].

The Return to Country Study, of which this methodological work is a part, aims to characterize the socioeconomic, environmental, health service, and biomedical factors driving the health outcomes and patterns of health service utilization experienced by First Nations Australians receiving kidney replacement therapy and investigate whether health service changes to address these identified barriers can achieve higher rates of kidney replacement therapy closer to home [23].

### Data Source and Management

The source of data for our motivating example is the Australia and New Zealand Dialysis and Transplant Registry (ANZDATA) [6,22]. ANZDATA receives, collates, and analyzes data from centers providing care for patients receiving long-term dialysis or kidney transplantation in Australia and New Zealand. Data submission is voluntary but complete. For this methodological study, we used the data extract provided by ANZDATA for the Return To Country Study (ANZRREQ-471) [23].

We received n=55,856 patient data on the course of treatments and patients’ history data from February 14, 1992, till December 31, 2023. Since our initial study period was defined from January 1, 2005, to December 31, 2023, we excluded patient data before January 01, 2005. This resulted in n=46,160 observations on the course of treatment and comorbidities data. With the revised study period definition (January 1, 2012–December 31, 2023), following consultation with a team of chief investigators, a total of 11,586 observations were excluded (n=44,270 individual level observations were retained out of 55,856). Due to 1743 missing observations for late referral, 808 on weight, and 188 on height variables, n=41,531 patient data were retained. In addition, for comparison purposes, centers were split into Indigenous and non-Indigenous centers. Some centers had fewer than 20 Indigenous patients. This required considering an adequate count of Indigenous patients per center for running the hierarchical logistic regression. Accordingly, centers with fewer than 20 Indigenous patients were excluded, which resulted in n=16,243 (25,288 observations deleted) individual-level data. Moreover, we dropped patients with missing postcode (2640 observations deleted), a total of n=13,603 remained. Finally, among the 13,603 observations, 3309 observations had censored status and hence were excluded. In addition, we excluded 55 missing observations on lung diseases, cardiovascular disease, and diabetes combined. Therefore, a total of 10,195 observations were included in our study.

In the following, we presented model specification, the derivation of the variance for the LogSIR using the delta method and a description of the bootstrap and Bayesian approaches for estimating variance of Log-SIR.

### Model Specification and Likelihood Definition

Since we have a binary outcome of receiving treatment close to home for end-stage kidney disease, denoted by *y_ci_*, from *n_c_* number of patients receiving treatment from center *c* for *N* centers, we proposed a Bernoulli sampling distribution for the probability of getting treatment close to home for the *i^th^* patient from center *c*. That is, *y_ci_* ∼ Bernoulli(*p_ci_*) and a random effects logistic regression model can be specified as:

$logit\left(p_{ci}\right)=\eta_{ci}=\beta_{0}+\beta_{1}X_{1ci}+\ldots +\beta_{k}X_{kci}+u_{c}$(1)

where *y_ci_* is the binary outcome for patient *i* in center *c*, *X*_1_*_ci,_ ... X_kci_* are *k* covariates for patient *i* in center *c*, *β*_0_*, β*_1_*, ..., β_k_* are fixed effects, *u_c_* is the random effect for center *c*, assumed to be normally distributed: $u_{c}∼N\left(0,\sigma_{c}^{2}\right)$, and *p*_ci_ =*P*(*y_ci_*=1).

We included the following covariates in our model: gender, age group, Indigenous status, lung disease, diabetes, BMI, cardiovascular disease, referral status, remoteness, and time period. And they were coded as follows: gender (male vs female categories), agegp (age group with 7 categories: ≥16‐26*,* ≥26‐36*,* ≥36‐46*,* ≥46‐56*,* ≥56‐66*,* ≥66‐76, and ≥76), Indigenous status (Indigenous vs non-Indigenous), lung (lung disease status: yes vs no), diabetes (diabetes status: yes vs no), late (late referral status: yes vs no), bmi30 (binary BMI status: BMI <30 kg/m^2^ vs BMI ≥30 kg/m^2^), mmm (Modified Monash Model remoteness scale with 7 categories: metropolitan areas [MM1], regional centers [MM2], large rural towns [MM3], medium rural towns [MM4], small rural towns [MM5], remote communities [MM6], and very remote communities [MM7]), and timegp (time periods: 2012‐2015, 2016‐2019, and 2020‐2023).

Accordingly, given *y_ci_* binary “Return to Country” outcome for individual *i* in center *c*, which is distributed as *y_ci_* ∼ Bernoulli(*p_ci_*), then the logit of the probability *p_ci_* is modeled as follows:


$$logit(p_{ci})=\beta_{0}+\beta_{1}\cdot gender_{ci}+\beta_{2}\cdot agegp_{ci}+\beta_{3}\cdot indigenous_{ci}+\beta_{4}\cdot lung_{ci}+\beta_{5}\cdot diabetes_{ci}+\beta_{6}\cdot cvd_{ci}+\beta_{7}\cdot late_{ci}+\beta_{8}\cdot bmi30_{ci}+\beta_{9}\cdot mmm_{ci}+\beta_{10}\cdot timegp_{ci}+centreid_{c}$$

where *β*_0_ is the global intercept, *β*_1_*, ...*, *β*_10_ are fixed-effect coefficients for the covariates, *centreid_c_* ∼ N(0*,σ_u_*^2^) is the group-level random intercept for center *c*, and *p_ci_*=Pr (*y_ci_*=1 | covariates).

Since we have individual-level data, we fitted a binary logistic regression model and computed the Log-SIR by aggregating: (1) the observed binary “Return to Home” status in center *c* and (2) the model-based predicted probabilities (used to calculate the expected number of patients returning home in center *c*).

Then, the Log-SIR is computed as:


$${\text{Log-SIR}}_{c}=\frac{\sum_{i\in c}y_{i}}{\sum_{i\in c}{\hat{p}}_{i}}$$

where *y_i_* ∈ {0*,*1} is the observed outcome for individual *i*, and $\hat{p}$*_i_* is the predicted probability of receiving treatment close to home for individual patient *i* from center *c*.

This approach is methodologically valid and commonly used in Bayesian hierarchical modeling and disease mapping, especially when individual-level data are available, but aggregate counts are not directly observed. Modeling binary outcomes using Bernoulli likelihoods (ie, logistic regression) is appropriate for estimating probabilities of outcome conditional on covariates. These estimated probabilities can then be summed within groups to yield expected counts for computing SIR or relative risks. This technique allows the derivation of SIR from model-based expected counts, which is consistent with the definition of indirect standardization [14,24-27]. Further details of the model specification can be found in Multimedia Appendix 1.

Application works using this approach include Kasza et al [28] and Normand et al [29]. Application of random intercept multilevel logistic regression models to indirectly standardize performance measures is explored by Clark and Moore [30] using National Trauma Data Bank data for the admission year 2008. Yang et al [31] explored hierarchical logistic regression (LR) modeling under various conditions applying Bayesian and frequentist methods.

### Delta Method for the Variance of the Log-SIR

The delta method is a technique used to approximate the variance of a function of 1 or more random variables [32-34]. The first-order Taylor series approximation for moments of ratio estimators is used to derive the mean and variance estimates; see Casella and Berger [32] (pages 244‐245). In the context of estimating the variance of the Log-SIR, we can apply the delta method to approximate the variance of $\log\left(\frac{O_{c}}{E_{c}}\right)$. It approximates the variance of a function of random variables by using the Jacobian matrix and the covariance matrix of the original variables; see Boos and Stefanski [35] (page 14). Accordingly, the variance of Log-SIR*_c_* is approximated by:


(2) $$\text{Var}\left({\text{Log-SIR}}_{c}\right)\approx \nabla g\cdot \text{Cov}\left(O_{c},E_{c}\right)\cdot \nabla g^{T}$$

where the covariance matrix of *O_c_* and *E_c_* is specified as:


$$\mathrm{Cov}(O_{c},E_{c})=\left(\begin{array}{cc} \mathrm{Var}(O_{c}) & \mathrm{Cov}(O_{c},E_{c})\\ \mathrm{Cov}(O_{c},E_{c}) & \mathrm{Var}(E_{c}) \end{array}\right)$$

And the Jacobian matrix (gradient) ∇*g* of the function *g*(*O_c_,E_c_*) with respect to *O_c_* and *E_c_* is given by: $\nabla g=\left(\begin{array}{cc} \frac{1}{O_{c}} & -\frac{1}{E_{c}} \end{array}\right)$

Substituting ∇*g* and Cov(*O_c_,E_c_*) into the formula, we get the final expression for the variance:

$\text{Var}\left(Log-{\text{SIR}}_{c}\right)\approx \frac{\text{Var}\left(O_{c}\right)}{O_{c}^{2}}+\frac{\text{Var}\left(E_{c}\right)}{E_{c}^{2}}-2\cdot \frac{\text{Cov}\left(O_{c},E_{c}\right)}{O_{c}E_{c}}$ (3)

Detailed derivation of the final formula for the variance of log(SIR) using the delta method given the model specification and the likelihood formulations above is presented in Multimedia Appendix 2.

The next section summarizes the estimates for Var(*O_c_*), Var(E*_c_*), and Cov(O*_c_,*E*_c_*).

Variance of O_c_: Var(O_c_)

Let *Y_i_* be the binary outcome for individual *i* in center *c*. The observed incidence *O_c_* is the sum of binary outcomes *Y_i_* for individuals within the *c^th^* center. If patients share hospital-level characteristics, the outcomes *Y_i_* are not independent but are correlated due to the shared random effect. The observed counts for center *c* are:


$$O_{c}=\sum_{i\in n_{c}}Y_{i}$$

The variance of *O_c_* is given by:


$$\text{Var}\left(O_{c}\right)=\text{Var}\left(\sum_{i\in n_{c}}Y_{i}\right)$$

Using the property of variance for the sum of random variables, this expands to:


$$\text{Var}\left(O_{c}\right)=\sum_{i\in n_{c}}\text{Var}\left(Y_{i}\right)+2\sum_{i§amp;lt;j\in n_{c}}\text{Cov}\left(Y_{i},Y_{j}\right)$$

This expression is derived from the formula for the variance of the sum of random variables. Here, Var(*Y_i_*) represents the variance of the individual observations, and Cov(*Y_i_,Y_j_*) is the covariance between pairs of observations. The factor of 2 in front of the covariance term accounts for the fact that each covariance term is counted only once when summing over pairs *i<j*.

For a logistic regression model with random intercepts, the variance and covariance terms are as follows:


(4) $$Var(Y_{i})=p_{i}\left(1-p_{i}\right)$$

(5) $$\text{Cov}\left(Y_{i},Y_{j}\right)=p_{i}\left(1-p_{i}\right)p_{j}\left(1-p_{j}\right)\sigma_{u}^{2}$$

(6) $$\text{Var}\left(O_{c}\right)=\sum_{i\in n_{c}}p_{i}\left(1-p_{i}\right)+2\sum_{i<j\in n_{c}}p_{i}\left(1-p_{i}\right)p_{j}\left(1-p_{j}\right)\sigma_{u}^{2}$$

### Derivation of Var(E)

The expected counts *E* are the sum of predicted probabilities *p_i_* for individuals within a center. The variance of *E* arises from the uncertainty in the predicted probabilities due to the random effects.

The expected counts for center *c* are:


$$E_{c}=\sum_{i\in n_{c}}p_{i}$$

The variance of *E_c_* is:


$$\text{Var}\left(E_{c}\right)=\sum_{i\in n_{c}}\text{Var}\left(p_{i}\right)+2\sum_{i§amp;lt;j\in n_{c}}\text{Cov}\left(p_{i},p_{j}\right)$$

For the random-effects logistic regression model:


$$\text{Var}\left(p_{i}\right)\approx {\left[p_{i}\left(1-p_{i}\right)\right]}^{2}\text{Var}\left(\eta_{i}\right)$$

where $\eta_{i}=x_{i}^{T}\beta +u_{c}$ is the linear predictor. The covariance between *p_i_* and *p_j_* (for *i*≠*j*) is as follows:


$$\text{Cov}\left(p_{i},p_{j}\right)\approx \left[p_{i}\left(1-p_{i}\right)\right]\left[p_{j}\left(1-p_{j}\right)\right]\text{Cov}\left(\eta_{i},\eta_{j}\right)$$

Since *η_i_* and *η_j_* share the same random effect *u_c_*:


$$\text{Cov}\left(\eta_{i},\eta_{j}\right)=\sigma_{u}^{2}$$

Thus:


$$\text{Cov}\left(p_{i},p_{j}\right)\approx \left[p_{i}\left(1-p_{i}\right)\right]\left[p_{j}\left(1-p_{j}\right)\right]\sigma_{u}^{2}$$

Combining these results:


$$\left[\text{Var}\left(E_{c}\right)=\sum_{i\in n_{c}}{\left[p_{i}\left(1-p_{i}\right)\right]}^{2}\text{Var}\left(\eta_{i}\right)+2\sum_{i<j\in n_{c}}\left[p_{i}\left(1-p_{i}\right)\right]\left[p_{j}\left(1-p_{j}\right)\right]\sigma_{u}^{2}\right]$$

### Derivation of Cov(O_c_, E_c_)

The covariance between *O_c_* and *E_c_*, where *O_c_* is the observed count and *E_c_* is the expected count for center *c*, arises because both depend on the same underlying probabilities *p_i_*, which are influenced by the shared random effect.

To derive the covariance Cov(*O_c_, E_c_*), given $\left(O_{c}=\sum_{i\in n_{c}}Y_{i}\right)$ (Observed count) and $\left(E_{c}=\sum_{i\in n_{c}}p_{i}\right)$ (Expected count), we have the covariance between *O_c_* and *E_c_* defined as:


$$\text{Cov}\left(O_{c},E_{c}\right)=\text{Cov}\left(\sum_{i\in n_{c}}Y_{i},\sum_{i\in n_{c}}p_{i}\right)$$

And using the property of covariance for sums, we get:


$$\text{Cov}\left(O_{c},E_{c}\right)=\sum_{i\in n_{c}}\text{Cov}\left(Y_{i},p_{i}\right)+2\sum_{i<j\in n_{c}}\text{Cov}\left(Y_{i},p_{j}\right)$$

Therefore, the final expression of Cov(*O_c_,E_c_*) becomes :

 $\text{Cov}\left(O_{c},E_{c}\right)=\sum_{i\in n_{c}}p_{i}\left(1-p_{i}\right)\text{Var}\left(\eta_{i}\right)+2\sum_{i§amp;lt;j\in n_{c}}p_{i}\left(1-p_{i}\right)p_{j}\left(1-p_{j}\right)\sigma_{u}^{2}$* *(8)

### Bootstrapping Approach

Commonly, the bootstrap approach is used to approximate variance of the log standardized incidence ratio. By sampling with replacement from the observed sample, creating a resampled dataset of size *n* and repeating this *B* times, it creates a nonparametric bootstrapped distribution [32], pages 479‐480. This distribution can be used to estimate the variance of the Log-SIR*_c_*. Mathematically, this can be summarized as:

 ${\hat{\sigma }}_{\text{Boot}}^{2}=\frac{1}{B-1}\sum_{b=1}^{B}{\left({\bar{\theta }}^{*}-{\hat{\theta }}_{b}^{*}\right)}^{2}$

with $\hat{\theta_{b}^{*}}$ the Log-SIR*_c_* value estimated in the *b^th^* bootstrap sample and $\overset{-}{\theta^{*}}$ the mean Log-SIR*_c_* estimated over the *B* bootstrap samples; here *B*=5000.

### Bayesian Approach

Given the model specification given in (1), the posterior distribution for a random effects logistic regression model can be expressed in a hierarchical form, integrating over the random effects *u_c_*. It can be recalled that the form of a posterior for hierarchical models is [35]:


$$\pi \left(\theta |Y=y\right)=\frac{f\left(y|\theta \right)\int \pi \left(\theta |\alpha \right)h\left(\alpha \right)d\alpha }{\int \int f\left(y|\theta \right)\pi \left(\theta |\alpha \right)h\left(\alpha \right)d\alpha d\theta }.$$

Using the likelihood for random effects logistic regression and priors for *β* and *u_c_*, the full posterior distribution can be shown to be:


(9) $$\begin{array}{l} \pi \left(\beta ,u|y,X\right)=\prod_{c=1}^{C}\prod_{i=1}^{n_{c}}{\left[\frac{1}{1+e^{-\left(x_{ci}^{⊤\beta }+u_{c}\right)}}\right]}^{y_{ci}}{\left[1-\frac{1}{1+e^{-\left(x_{ci}^{⊤\beta }+u_{c}\right)}}\right]}^{1-y_{ci}}\times \frac{1}{\sqrt{{\left(2\pi \right)}^{p}\left|\Sigma_{\beta }\right|}}\exp\left(-\frac{1}{2}{\left(\beta -\mu_{\beta }\right)}^{⊤\Sigma_{\beta }^{-1}}\left(\beta \;-\mu_{\beta }\right)\right)\times \prod_{c=1}^{C}\frac{1}{\sqrt{2\pi \sigma_{u}^{2}}}\exp\left(-\frac{u_{c}^{2}}{2\sigma_{u}^{2}}\right) \end{array}$$

Details of the derivation of the full posterior distribution are summarized in Multimedia Appendix 3.

Due to the need to integrate out the nuisance parameters in (9) and lack of conjugate priors, and the hierarchy involved, computing difficult integrals is required using MCMC methods whereby a dependent sequence of random variables is obtained with the property that in the limit these random variables have the posterior distribution.

Accordingly, the following information is used to estimate the variance of the Log-SIR using the Bayesian approach:

*y_ci_* ∼ Bernoulli(*p_ci_*)


$$\text{logit}\left(p_{ci}=P\left(y_{ci}=1\right)\right)=\eta_{ci}=\beta_{0}+\sum_{m=1}^{k}\beta_{m}X_{mci}+u_{c}$$

where:

$\left(\beta_{0},\beta_{1},\ldots ,\beta_{k}∼N\left(0,\sigma_{c}^{2}\right)\right),\left(u_{c}∼N\left(0,\sigma_{c}^{2}\right)\right),\left(\sigma_{c}^{2}=\frac{1}{\tau }\right)$, and *τ* ∼ Gamma(0.001*,*0.001).

The MCMC simulation is conducted using 25,500 iterations with 500 initial burn-ins, 3 chains, and a single thinning interval. Analysis was performed using the R Statistical Programming Language and the associated R packages [36-42].

### Performance Metrics: Bias, Variance, and MSE

To compare the performance of the delta method, bootstrap, and MCMC approaches for estimating the variance of the Log-SIR, we evaluated several criteria such as bias (the difference between the expected value of the estimator and the true value), consistency (the estimator should converge to the true value as the sample size increases), and MSE (for overall accuracy).

### Ethical Considerations

Ethical approval was obtained from the Human Research Ethics Committee (HREC) of the Northern Territory Department of Health and Menzies School of Health Research (2019‐3530), Far North Queensland HREC (2023/QCH/99606 (Nov ver 4)‐1732), the Central Adelaide Local Health Network HREC (2023/HRE00209), the Aboriginal Health Council of South Australia (AHREC Protocol number 04-23-1078), the Aboriginal Health and Medical Research Council of New South Wales (AH&MRC HREC reference: 2230/24), and the Far North Queensland Human Research Ethics Committee (FNQ HREC reference: HREC/2023/QCH/99606 (Nov ver 4)‐1732). For information on informed consent details, please refer to our protocol paper on the “Return to Country” project, which can be accessed here [23].

## Results

### Variance of Log-SIR Using the 3 Estimation Methods

A summary of bias, along with variance and MSE, is shown in Table 1.

**Table 1.** Comparison of bias, variance, and mean squared error for different estimation methods.

Method	Bias	Variance	Mean squared error
Delta	0.01927454	1.696437e-04	0.0005411516
Bootstrap	0.02566281	2.771867e-04	0.0009357665
Bayesian	0.01922758	5.142122e-05	0.0004211210

The analysis result indicated that the bias values across all methods were similar, with MCMC slightly showing the lowest bias (0.01922), followed by the delta method (0.01927) and the bootstrap method (0.02567), respectively. This suggests that the Bayesian MCMC method provides a slightly less biased variance estimate of Log-SIR than the other methods. In addition, the Bayesian MCMC method exhibits the lowest variance (0.00005), indicating more stable and less dispersed estimates of the Log-SIR. Higher variance was observed in the delta method (0.00016), while the bootstrapping approach resulted in the highest variance (0.00027), introducing more uncertainty in the Log-SIR estimates. Looking at the overall accuracy of the methods, the Bayesian MCMC method had the lowest MSE (0.00042), indicating better overall accuracy. The delta method follows with an MSE of 0.00054, and the bootstrap method had the highest MSE (0.00094), showing it to be the least reliable method among the methods compared.

The result, in general, indicated lower values on bias, variance, and MSE values. Lower bias values indicated that the estimators are more accurate on average, lower variance indicated that the estimators are more consistent, and lower MSE indicated that the estimators are both accurate and consistent. However, the parameter estimates were the lowest for the MCMC method, indicating the Bayesian approach to be a more preferred approach for the estimation of the variance of the Log-SIR (var[Log-SIR]). MCMC is the best-performing method as it has the lowest bias, variance, and MSE. The delta method performs reasonably well but has slightly higher variance and MSE than MCMC. Bootstrap captures variability well but introduces more uncertainty, as seen in its high variance and MSE.

**Figure 1.**
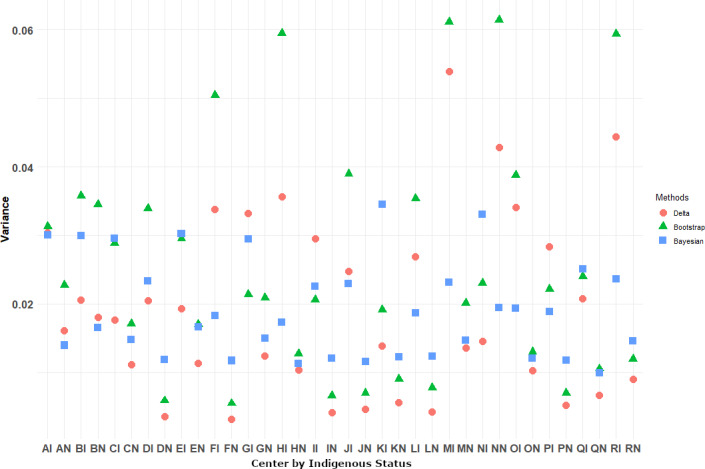
Delta method, bootstrapping, and Bayesian approaches.

In addition, a comparison of the 3 methods in terms of consistency is shown in Figure 1. Accordingly, Figure 1 highlights the trade-offs among the variance estimation methods. While bootstrapping tends to be more variable, MCMC provides more stable estimates, and the delta method offers computational efficiency but can be less precise. Bootstrapping (green) shows higher variance. The green points, representing bootstrap-based variance estimates, are often higher compared to the other 2 methods. This suggests that bootstrapping introduces additional variability, which is expected since it resamples the data and can exaggerate variance in small samples.

However, the Bayesian MCMC estimates (the blue points) are more stable. They are generally lower than bootstrapping but slightly higher than the delta method for most of the cases. The Bayesian methods incorporate prior information, and this leads to more stabilized variance estimates.

The delta method (red) is the most conservative and hence it often yields the lowest variance estimates. This method uses first-order approximations and may underestimate variance, especially for complex or skewed data distributions.

A summary table for each center is shown in Table 2. As is evident from Table 2, the standard errors were highly variable across centers using the bootstrap method followed by the delta method.

**Table 2.** Comparison of delta, bootstrap method, and Bayesian estimates along with 95% coverage by center.^a^

Center	Mean	SE	LCI^b^	UCI^c^	Mean	SE	LCI	UCI	Mean	SE	LCrI^d^	UCrI^e^
AI	0.100	0.030	0.041	0.159	0.016	0.031	−0.050	0.071	0.031	0.030	−0.019	0.098
AN	0.010	0.016	−0.021	0.041	0.028	0.023	−0.019	0.069	−0.017	0.014	−0.038	0.016
BI	−0.382	0.021	−0.423	−0.341	−0.453	0.036	−0.525	−0.386	−0.441	0.030	−0.491	−0.374
BN	−0.100	0.018	−0.135	−0.065	−0.092	0.035	−0.164	−0.027	−0.131	0.017	−0.156	−0.093
CI	−0.148	0.018	−0.183	−0.113	−0.220	0.029	−0.277	−0.165	−0.209	0.030	−0.258	−0.142
CN	−0.008	0.011	−0.030	0.014	0.009	0.017	−0.026	0.041	−0.034	0.015	−0.057	0.000
DI	−0.090	0.020	−0.129	−0.051	−0.130	0.034	−0.200	−0.068	−0.139	0.023	−0.177	−0.086
DN	−0.017	0.004	−0.025	−0.009	0.026	0.006	0.014	0.037	−0.025	0.012	−0.043	0.003
EI	−0.057	0.019	−0.094	−0.020	−0.135	0.030	−0.197	−0.080	−0.122	0.030	−0.172	−0.054
EN	−0.008	0.011	−0.030	0.014	0.000	0.017	−0.034	0.032	−0.038	0.017	−0.063	0.001
FI	−0.004	0.034	-0.071	0.063	-0.012	0.050	-0.125	0.073	-0.040	0.018	-0.069	0.002
FN	-0.021	0.003	-0.027	-0.015	0.025	0.005	0.015	0.036	-0.027	0.012	-0.044	0.001
GI	0.143	0.033	0.078	0.208	0.066	0.021	0.018	0.102	0.075	0.029	0.027	0.142
GN	−0.029	0.012	−0.053	−0.005	−0.014	0.021	−0.057	0.024	−0.057	0.015	−0.080	−0.022
HI	−0.038	0.036	−0.109	0.033	−0.044	0.060	−0.174	0.059	−0.075	0.017	−0.103	−0.035
HN	0.020	0.010	0.000	0.040	0.054	0.013	0.027	0.077	0.000	0.011	−0.017	0.027
II	0.103	0.029	0.046	0.160	0.067	0.021	0.021	0.101	0.053	0.023	0.017	0.104
IN	−0.008	0.004	−0.016	0.000	0.032	0.007	0.018	0.044	−0.019	0.012	−0.038	0.009
JI	−0.033	0.025	−0.082	0.016	−0.075	0.039	−0.157	−0.005	−0.084	0.023	−0.122	−0.033
JN	−0.002	0.005	−0.012	0.008	0.038	0.007	0.024	0.051	−0.015	0.012	−0.032	0.013
KI	0.015	0.014	−0.012	0.042	−0.085	0.019	−0.125	−0.048	−0.055	0.035	−0.113	0.021
KN	−0.015	0.006	−0.027	−0.003	0.020	0.009	0.002	0.038	−0.030	0.012	−0.049	−0.002
LI	0.019	0.027	−0.034	0.072	0.008	0.035	−0.068	0.069	−0.019	0.019	−0.049	0.023
LN	−0.032	0.004	−0.040	−0.024	0.008	0.008	−0.008	0.022	−0.043	0.012	−0.061	−0.013
MI	0.068	0.054	−0.038	0.174	0.028	0.061	−0.113	0.116	0.017	0.023	−0.021	0.070
MN	0.000	0.014	−0.027	0.027	0.015	0.020	−0.027	0.052	−0.028	0.015	−0.050	0.006
NI	−0.111	0.014	−0.138	−0.084	−0.195	0.023	−0.241	−0.153	−0.176	0.033	−0.230	−0.102
NN	0.011	0.043	−0.073	0.095	0.003	0.061	−0.139	0.099	−0.027	0.019	−0.057	0.018
OI	0.046	0.034	−0.021	0.113	0.032	0.039	−0.052	0.097	0.007	0.019	−0.024	0.051
ON	0.017	0.010	−0.003	0.037	0.048	0.013	0.020	0.072	−0.004	0.012	−0.022	0.025
PI	0.077	0.028	0.022	0.132	0.065	0.022	0.016	0.102	0.039	0.019	0.008	0.082
PN	0.010	0.005	0.000	0.020	0.047	0.007	0.033	0.060	−0.004	0.012	−0.022	0.023
QI	0.062	0.021	0.021	0.103	0.012	0.024	−0.040	0.055	0.008	0.025	−0.033	0.065
QN	−0.006	0.007	−0.020	0.008	0.037	0.011	0.015	0.056	−0.020	0.010	−0.035	0.003
RI	0.021	0.044	−0.065	0.107	−0.014	0.059	−0.151	0.085	−0.026	0.024	−0.064	0.027
RN	0.014	0.009	−0.004	0.032	0.035	0.012	0.010	0.057	−0.009	0.015	−0.031	0.025

a All units are on the natural log scale.

b LCI: 95% lower confidence limit.

c UCL: 95% upper confidence limit.

d LCrI: 95% lower credible interval.

e UCrI: 95% upper credible interval.

In summary, there are notable variations in variance estimates across centers. Some centers exhibit more spread between methods, suggesting that the choice of method affects variance estimates significantly.

Similarly, a summary table of false discovery rates (FDRs) for each center is shown in Table 3. It is evident that there are notable variations in FDR estimates across centers. Some centers exhibit more spread between methods, suggesting that the choice of method affects variance and hence the resulting coverage significantly.

**Table 3.** Comparison of delta, bootstrap, and Bayesian estimates along with 95% false discovery rates by center.

Center	Mean	SE	LFDR^a^	UFDR^b^	Mean	SE	LFDR	UFDR	Mean	SE	LFDR	UFDR
AI	0.100	0.030	−0.059	0.059	0.016	0.031	−0.061	0.061	0.031	0.030	−0.059	0.059
AN	0.010	0.016	−0.032	0.032	0.028	0.023	−0.045	0.045	−0.017	0.014	−0.027	0.027
BI	−0.382	0.021	−0.040	0.040	−0.453	0.036	−0.070	0.070	−0.441	0.030	−0.059	0.059
BN	−0.100	0.018	−0.035	0.035	−0.092	0.035	−0.068	0.068	−0.131	0.017	−0.032	0.032
CI	−0.148	0.018	−0.035	0.035	−0.220	0.029	−0.057	0.057	−0.209	0.030	−0.058	0.058
CN	−0.008	0.011	−0.022	0.022	0.009	0.017	−0.034	0.034	−0.034	0.015	−0.029	0.029
DI	−0.090	0.020	−0.040	0.040	−0.130	0.034	−0.067	0.067	−0.139	0.023	−0.046	0.046
DN	−0.017	0.004	−0.007	0.007	0.026	0.006	−0.012	0.012	−0.025	0.012	−0.023	0.023
EI	−0.057	0.019	−0.038	0.038	−0.135	0.030	−0.058	0.058	−0.122	0.030	−0.059	0.059
EN	−0.008	0.011	−0.022	0.022	0.000	0.017	−0.033	0.033	−0.038	0.017	−0.033	0.033
FI	−0.004	0.034	−0.066	0.066	−0.012	0.050	−0.099	0.099	−0.040	0.018	−0.036	0.036
FN	−0.021	0.003	−0.006	0.006	0.025	0.005	−0.011	0.011	−0.027	0.012	−0.023	0.023
GI	0.143	0.033	−0.065	0.065	0.066	0.021	−0.042	0.042	0.075	0.029	−0.058	0.058
GN	-0.029	0.012	−0.024	0.024	−0.014	0.021	−0.041	0.041	−0.057	0.015	−0.029	0.029
HI	−0.038	0.036	−0.070	0.070	−0.044	0.060	−0.117	0.117	−0.075	0.017	−0.034	0.034
HN	0.020	0.010	−0.020	0.020	0.054	0.013	−0.025	0.025	0.000	0.011	−0.022	0.022
II	0.103	0.029	−0.058	0.058	0.067	0.021	−0.040	0.040	0.053	0.023	−0.044	0.044
IN	−0.008	0.004	−0.008	0.008	0.032	0.007	−0.013	0.013	−0.019	0.012	−0.024	0.024
JI	−0.033	0.025	−0.048	0.048	−0.075	0.039	−0.076	0.076	−0.084	0.023	−0.045	0.045
JN	−0.002	0.005	−0.009	0.009	0.038	0.007	−0.014	0.014	−0.015	0.012	−0.023	0.023
KI	0.015	0.014	−0.027	0.027	−0.085	0.019	−0.038	0.038	−0.055	0.035	−0.068	0.068
KN	−0.015	0.006	−0.011	0.011	0.020	0.009	−0.018	0.018	−0.030	0.012	−0.024	0.024
LI	0.019	0.027	−0.053	0.053	0.008	0.035	−0.069	0.069	−0.019	0.019	−0.037	0.037
LN	−0.032	0.004	−0.008	0.008	0.008	0.008	−0.015	0.015	−0.043	0.012	−0.024	0.024
MI	0.068	0.054	−0.106	0.106	0.028	0.061	−0.120	0.120	0.017	0.023	−0.045	0.045
MN	0.000	0.014	−0.027	0.027	0.015	0.020	−0.039	0.039	−0.028	0.015	−0.029	0.029
NI	−0.111	0.014	−0.028	0.028	−0.195	0.023	−0.045	0.045	−0.176	0.033	−0.065	0.065
NN	0.011	0.043	−0.084	0.084	0.003	0.061	−0.120	0.120	−0.027	0.019	−0.038	0.038
OI	0.046	0.034	−0.067	0.067	0.032	0.039	−0.076	0.076	0.007	0.019	−0.038	0.038
ON	0.017	0.010	−0.020	0.020	0.048	0.013	−0.026	0.026	−0.004	0.012	−0.024	0.024
PI	0.077	0.028	−0.056	0.056	0.065	0.022	−0.043	0.043	0.039	0.019	−0.037	0.037
PN	0.010	0.005	−0.010	0.010	0.047	0.007	−0.014	0.014	−0.004	0.012	−0.023	0.023
QI	0.062	0.021	−0.041	0.041	0.012	0.024	−0.047	0.047	0.008	0.025	−0.049	0.049
QN	−0.006	0.007	−0.013	0.013	0.037	0.011	−0.021	0.021	−0.020	0.010	−0.019	0.019
RI	0.021	0.044	−0.087	0.087	−0.014	0.059	−0.116	0.116	−0.026	0.024	−0.046	0.046
RN	0.014	0.009	−0.018	0.018	0.035	0.012	−0.023	0.023	−0.009	0.015	−0.029	0.029

a LFDR: 95% lower false discovery rate.

b UFDR: 95% upper false discovery rate.

In the next section, we presented funnel plots constructed using the 3 methods for assessing centers’ performance in providing services close to home for patients with end-stage kidney disease. The focus is to highlight how the variance estimation methods provide somewhat variable plots and how they affect interpretation and decision-making on the performance of centers in service provision.

### Centers’ Performance Using Funnel Plots

**Figure 2.**
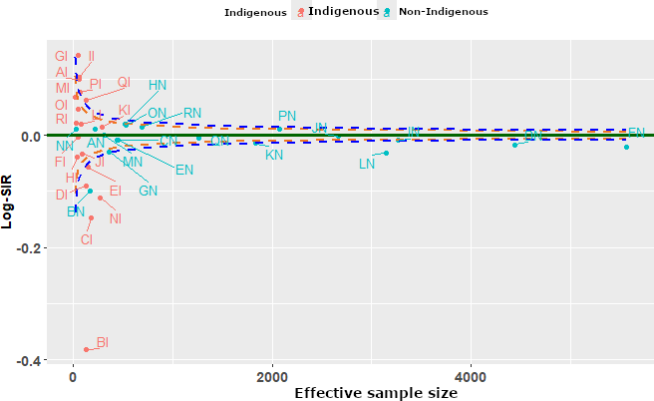
Funnel plot using the delta method. Log-SIR: logarithm of the standardized incidence ratio.

**Figure 3.**
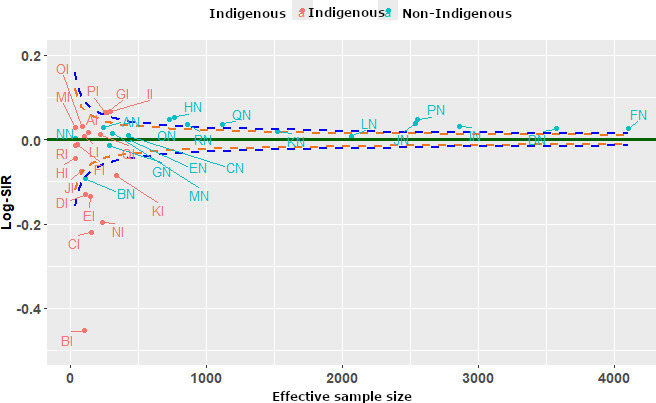
Funnel plot using the bootstrapping method. Log-SIR: logarithm of the standardized incidence ratio.

**Figure 4.**
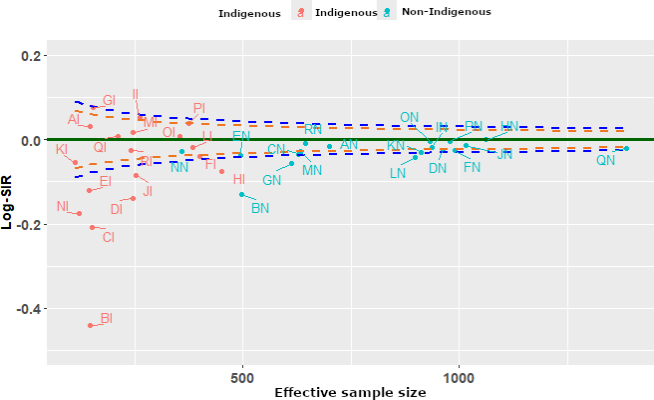
Funnel plot using the Bayesian Markov chain Monte Carlo method. Log-SIR: logarithm of the standardized incidence ratio.

A summary funnel plot using the 3 methods is displayed in Figures 2-4. Each funnel plot has different variance estimates for the same underlying data. The funnel plots evaluate center-level performance in treating patients with end-stage kidney disease close to home by comparing the Log-SIR across different centers stratified by Indigenous status. The x-axis represents effective sample size (defined as a measure of the variability of the Log-SIRs for each center relative to the total variability of all Log-SMRs [28,28]), while the y-axis measures Log-SIR, indicating whether observed rates of receiving treatment close to home are higher or lower than expected. Centers within the upper and lower FDRs indicate expected performance in treating patients close to home (are in the region of average performance). The dashed lines forming funnels around the horizontal solid line (Log-SIR=0) indicate expected variation, with centers falling outside these limits exhibiting statistically significant differences from the norm.

Figure 2 presents a funnel plot that compares the performance of centers using the delta method for estimating the variance of the Log-SIR. Centers within the upper and lower FDRs indicate expected performance in treating patients close to home (are in the region of average performance). The dashed lines forming funnels around the horizontal solid line (Log-SIR=0) indicate expected variation, with centers falling outside these limits exhibiting statistically significant differences from the norm.

Using this approach, 6 centers, BI, BN, CI, DI, EI, and NI, were low performing, while 3 centers, GI, II, and QI, were higher-than-average performers. The remaining centers lie within the FDRs, being average performers in treating patients close to home. Center BI shows the lowest Log-SIR, suggesting exceptionally lower performance in treating patients close to home. Overall, larger centers exhibit more stable Log-SIR values, while smaller centers experience greater variation, reinforcing the importance of center size in the assessment of centers’ performance in treating patients close to home. Using this method, the variance of Log-SIR appears relatively low, with most values concentrated around zero. Some extreme values (outliers) are present on the left-hand side, indicating a few centers with more deviation. The spread of points suggests that this method results in a tighter distribution of Log-SIR.

Figure 3 compares centers using the bootstrapping approach. Using this method, 7 centers, BI, BN, CI, DI, EI, NI, and KI, were lower-than-average performers. However, 12 centers, GI, II, PI, DN, FN, HN, IN, JN, ON, PN, QN, and RN, were found to be higher-than-average performing. The remaining centers lie within the FDRs, being average performers in treating patients close to home. Notably, BI remains an outlier with the lowest Log-SIR, reflecting exceptionally low performance in treating patients close to home. Using bootstrap, the variance is slightly larger compared with the first plot. The spread of Log-SIR values is more noticeable, with a wider range of deviations from zero. More centers have larger deviations, particularly on the left side, compared with the delta method.

Figure 4 presents a funnel plot that compares the performance of centers using the Bayesian approach for estimating the variance of the Log-SIR. Accordingly, 11 centers, BI, BN, CI, DI, EI, GN, HI, JI, KI, NI, and LN, were found low-than-expected performers, and no center was found to be top performing in treating patients close to home. Larger centers exhibit more stable Log-SIR values, reinforcing the reliability of their performance assessments. Using the Bayesian approach, the variance of Log-SIR is still larger than the first plot but somewhat comparable with the second. The spread is not as extreme as in the second plot, but it still shows noticeable deviations. There are clear differences in the spread of values across regions.

The delta method results in the least variance in Log-SIR, while the bootstrapping method has the highest variance, with a wider spread of values. Clearly, the Bayesian approach has an intermediate variance, showing more spread than the first method but less than the second.

## Discussion

### Overview

Our study results highlight center-level differences in treating patients close to home, and this is coupled with variability in variance estimation by the 3 methods. The stability of the Log-SIR using the Bayesian approach may be due to the method borrowing strength from prior beliefs, which are summarized using probability distributions that smooth variability in estimation.

In health care providers’ performance assessment, standardized incidence ratios (SIRs) and standardized mortality ratios (SMRs) are essential tools used to assess whether observed rates of disease or death deviate from what is expected. Accurate estimation of variance in these ratios is crucial as it affects decision-making regarding providers’ performance, resource allocation, and quality improvement strategies. In this study, we compared 3 methods, namely, the delta method, bootstrapping, and Bayesian approach, to estimate the variance of the Log-SIR given by equation 3 and considered funnel plot approaches to build FDRs around the Log-SIR using these 3 variance estimators. The variance estimation methods have been widely discussed in statistical literature. Gelman et al [43] emphasize that Bayesian methods, particularly MCMC, provide more stable estimates due to their ability to incorporate prior information and reduce uncertainty. Similarly, Efron and Tibshirani [11] discuss bootstrapping as a flexible but sometimes overly variable approach, which aligns with our findings of increased variance in bootstrapped estimates.

The delta method is frequently used in epidemiology for variance estimation [44]. It provides an efficient and straightforward way of estimating the variance of Log-SIR or Log-SMR, especially when the distribution of the underlying data was correctly specified. This method can be computationally efficient, but its accuracy may suffer in cases where the underlying distribution deviates significantly from the assumed form [45]. When applied in health care decision-making, such as assessing the performance of hospitals based on SMRs, the delta method may underestimate variance if assumptions are violated. This could lead to incorrect conclusions regarding the performance of health care providers.

Variance estimation using the delta method for metrics other than SMR has been used intensively. For instance, Normand and Shahian [46] applied the delta method to approximate the variance of demographic parameters in avian biology studies. Although not directly related to health care, this study illustrates the broader applicability of the delta method in estimating variances of complex ratios. Also, Lee et al [47] compared the Green, delta, and Monte Carlo methods for calculating the 95% CI for population-attributable fraction. In addition, Sauer et al [48] applied the delta method for variance estimation for effective coverage measures. There is limited study that directly applied the delta method in the estimation of Log-SIR used in the assessing performance of health care providers in the provision of health services for a given outcome.

Bootstrapping, on the contrary, has the advantage of not relying on distributional assumptions and can be used to directly estimate the distribution of Log-SIR or Log-SMR. This can lead to more robust variance estimates, particularly in settings with small sample sizes or unknown distributions. By resampling, bootstrapping accounts for sampling variability and can help improve the precision of performance assessments [11]. For instance, Kasza et al [28] used bootstrapping for evaluating the performance of Australian and New Zealand intensive care units in 2009 and 2010 quantified by the standardized mortality ratio. Moreover, Walters and Campbell [49] used bootstrap methods for analyzing health-related quality-of-life outcomes used in clinical trials as primary outcome measures. They found that certain bootstrap methods provided more accurate variance estimates, especially when the distribution of the outcome is unknown or ordinal scale.

By contrast, Bayesian methods provide a full posterior distribution for variance estimates, allowing for the incorporation of prior knowledge, such as expert opinion or historical data on hospital performance. This can lead to more flexible and informative variance estimation, especially when data are sparse or prior knowledge is available. Bayesian methods can also be used to model hierarchical structures (eg, hospitals within regions), providing more precise estimates of performance at various levels [32].

A study by George et al [50] applied Bayesian hierarchical models to estimate hospital performance in the Hospital Compare model for acute myocardial infarction mortality. They found that indirect standardization fails to adequately control for differences in patient risk factors and systematically underestimates mortality rates at the low-volume hospitals.

Below, we have summarized the variability in variance estimates and their implications on funnel plots and epidemiological studies.

### Variability in Log SIR Variance Estimates

The 3 methods yield different variance estimates for the same underlying data. Bootstrapping tends to produce higher variance estimates due to the nature of resampling, which can exaggerate variability, particularly in small samples [51]. By contrast, Bayesian (MCMC) estimates tend to be more stable, benefiting from prior distributions that help regularize estimates, a characteristic also observed in Bayesian hierarchical models for disease mapping [52]. The delta method, being a first-order approximation, is the most conservative, often producing the lowest variance estimates, which may lead to underestimation in complex data structures [32]. These differences highlight the importance of choosing an estimation method suited to the underlying data characteristics and sample size.

In our study, the variance estimates differ across methods, with bootstrapping tending to show more extreme values (both high and low) compared to the other 2 methods. MCMC appears to provide more stable and generally lower variance estimates compared to bootstrapping. The delta method is relatively consistent but tends to lie between the MCMC and bootstrap estimates. Some centers have noticeably higher variance estimates for all 3 methods (eg, locations where green dots are well above the others). This suggests that uncertainty in Log-SIR estimation varies by center, possibly due to differences in sample size, population characteristics, or underlying risk factors. Bootstrapping shows more variability, which is expected since it resamples data and may amplify variability in small samples. MCMC provides more stable estimates, benefiting from Bayesian shrinkage and prior information incorporation. The delta method is computationally efficient but may underestimate variance in some cases (eg, when normality assumptions are violated) [53]. Centers with higher variance estimates (especially under bootstrapping) suggest that Log-SIR estimates are more uncertain there, which should be considered when making public health decisions. If variance estimates are too high, it may indicate the need for larger sample sizes or improved data collection in those centers.

### Impact on Funnel Plots

The funnel plots illustrate how these methods influence the distribution of Log-SIR estimates. The Bayesian approach exhibits a more stabilized pattern, particularly at smaller sample sizes, where shrinkage effects help reduce extreme values. This aligns with findings from Spiegelhalter et al [54], who demonstrated that Bayesian hierarchical modeling effectively mitigates overdispersion in epidemiological data. Conversely, the bootstrapping approach results in greater spread at smaller sample sizes, reflecting its sensitivity to sample fluctuations. Similar findings have been reported in comparative studies on variance estimation methods, where bootstrapping is noted to introduce greater variability but remains valuable for robust uncertainty estimation [11]. Although both methods show convergence of Log-SIR estimates toward zero as sample sizes increase, bootstrapping maintains slightly higher variance, reinforcing the need for careful interpretation in small-sample studies.

### Implications for Epidemiological Studies

The choice of variance estimation method has significant implications for epidemiological research. Bayesian methods offer improved stability and are particularly useful when incorporating prior knowledge is beneficial. Studies have shown that Bayesian approaches reduce estimation bias and enhance interpretability in spatial epidemiology [55]. Bootstrapping, despite its higher variability, remains a valuable tool for robust uncertainty estimation, especially when parametric assumptions may not hold [56]. Meanwhile, the delta method, though computationally simple, may underestimate variance, making it less reliable for complex data scenarios, as previously noted in statistical inference literature [32]. These findings align with broader discussions on variance estimation in epidemiology, emphasizing the trade-offs between robustness, computational efficiency, and precision [57].

### Principal Findings

These findings highlight the importance of selecting an appropriate variance estimation method depending on the study context. Bayesian methods may be preferable when stability and regularization are critical, while bootstrapping is useful for assessing variability in more flexible settings. The delta method should be used cautiously, particularly when dealing with skewed or complex distributions. Future research should explore hybrid approaches that combine the strengths of these methods for more robust inference [11,32].

Our results showed that Bayesian approaches provided more conservative estimates with tighter credible intervals, particularly in hospitals with small case volumes. We demonstrated that Bayesian MCMC outperforms the other methods in terms of lower variance and MSE, making it the preferred choice for estimating Log-SIR variance when computational resources permit.

### Limitations

Our study has several limitations. First, while understanding the differences between variance estimation methods is crucial for assessing the reliability of SIR estimates across different centers, we did not consider how model choice influences variance estimates and hence the resulting statistical inference. That is, we only used hierarchical logistic regression model for modeling the binary individual-level outcome. Therefore, we did not explore the implication of using the Poisson model for aggregated data on the resulting variance estimates using the 3 methods. Second, we considered only nonparametric bootstrapping, and the implications of parametric bootstrapping were not assessed. Third, we did not consider other transformations than logarithmic transformations and their effects on the interpretation of providers’ performance. For instance, Quaresma et al [12] investigated the implications of identity(log), complementary log-log, logit, and logarithmic transformation in their study of cancer survival. Finally, within the random effects logistic regression, we considered only logit link, and other links such as probit and complementary log-log link were not considered here.

### Conclusions

In conclusion, the choice of variance estimation method plays a significant role in how health care providers’ performance is assessed. While each method has its strengths and weaknesses, bootstrapping and Bayesian approaches generally provide more reliable estimates of uncertainty compared to the delta method. However, the choice of method should consider computational resources, data structure, and the available prior knowledge for Bayesian methods. Decision-makers should be aware of the implications of variance estimation on conclusions regarding provider performance, which can influence policy, resource allocation, and quality improvement initiatives in health care settings. In terms of decision-making, the choice of variance estimation method can affect the conclusions drawn about the performance of health care providers. Using the delta method may lead to an underestimation of uncertainty, especially when the data do not meet distributional assumptions. Bootstrapping, while more robust, may be computationally intensive, especially with large datasets. Bayesian methods, with their flexibility and ability to incorporate prior knowledge, can be powerful tools but require careful specification of priors and may be computationally demanding.

## Supplementary material

10.2196/77415 Multimedia Appendix 1 Model and logarithm of standardized incidence ratio definitions.

10.2196/77415 Multimedia Appendix 2 Delta method.

10.2196/77415 Multimedia Appendix 3 Bayesian approach.

## Abbreviations

ANZDATA: Australia and New Zealand Dialysis and Transplant Registry
FDR: false discovery rate
HREC: Human Research Ethics Committee
Log: logarithm
Log-SIR: logarithm of the standardized incidence ratio
MCMC: Markov chain Monte Carlo
MSE: mean squared error
SIR: standardized incidence ratio
SMR: standardized mortality ratio
