# Supplementary material for: Estimating Variance of Log Standardized Incidence Ratios Assessing Health Care Providers’ Performance: Comparative Analysis Using Bayesian, Bootstrap, and Delta Method Approaches
Source: JMIRx Med. 2025 Oct 9;6:e77415. doi: 10.2196/77415 (PMC12605305; doi:10.2196/77415)
Supplement: Multimedia Appendix 1 [file xmed-v6-e77415-s001.docx]

# Appendix A. Model and log SIR Definitions

In the following we presented model specification, the derivation of the variance for the Log-SIR using the delta-method, and a description of the bootstrap and Bayesian approaches for estimating variance of Log-SIR.

## Model specification

Since we have binary outcome of receiving treatment close to home for ESKD, denoted by *y_ci_*, from *n_c_* number of patients receiving treatment from the *c^th^* centre/hospital. Where, *c, ..., N* centres, we proposed a Bernoulli sampling distribution for the probability of getting treatment close to home for the *i^th^* patient from the *c^th^* centre.

That is, *y_ci_* ∼ Bernoulli(*p_ci_*) and a random effects logistic regression model can be specified as:

$logit\left( p_{ci} \right)=\eta_{ci}=\beta_{0}+\beta_{1}X_{1ci}+\ldots+\beta_{k}X_{kci}+u_{c}$ (1)

where, *y_ci_* is the binary outcome for patient *i* in centre *c*, *X*_1_*_ij_ ...X_kij_* are *k* covariates for patient *i* in centre *c*, *β*_0_*, β*_1_*, ..., β_k_* are fixed effects, and *u_c_* is the random effect for centre *c*, assumed to be normally distributed: $u_{c}\sim\mathcal{N}\left( 0,\sigma_{c}^{2} \right)$. That is, in a random effects model, the random effects *u_c_* are assumed to follow a normal distribution: $u_{c}\sim\mathcal{N}\left( 0,\sigma_{c}^{2} \right)$.

The density function for the normal distribution is:

$$f\left( u_{c} \right)=\frac{1}{\sqrt{2\pi\sigma_{c}^{2}}}\exp\left( -\frac{u_{c}^{2}}{2\sigma_{c}^{2}} \right)$$

The probability of observing *y_ci_* given the fixed and random effects is:

$$P\left( y_{ci} | \beta,u_{c} \right)=\frac{e^{y_{ci}\eta_{ci}}}{1+e^{\eta_{ci}}}$$

That is, $P\left( y_{ci}=1 | \beta,u_{c} \right)=\frac{e^{\eta_{ci}}}{1+e^{\eta_{ci}}}$ and $P\left( y_{ci}=0 | \beta,u_{c} \right)=\frac{1}{1+e^{\eta_{ci}}}$

where $\eta_{ci}=\beta_{0}+\beta_{1}X_{1ci}+\ldots+\beta_{k}X_{kci}+u_{c}$is the linear predictor.

The probability for a single patient *i* in centre *c* is:

$$P\left( y_{ci} | \beta,u_{c} \right)=\left( \frac{e^{\eta_{ci}}}{1+e^{\eta_{ci}}} \right)^{y_{ci}}\left( \frac{1}{1+e^{\eta_{ci}}} \right)^{1-y_{ci}}$$

The fixed effects *β* and the variance parameter *σ_u_*^2^ are estimated using maximum likelihood estimation (MLE) through the ‘glmer‘ function in R.

## Standardized Incidence Ratio (SIR)

In the next, we presented details of the derivation of the final formula for the variance of log(SIR) using the delta method given the sampling distribution and the model formulations above.

The SIR for the *c^th^* centre is defined as:

$$\text{SIR}_{c}=\frac{O_{c}}{E_{c}}$$

Where *O_c_* is the sum of the observed outcomes (receiving treatment close to home) for all patients

in centre *c*:

$$O_{c}=\sum_{i=1}^{n_{c}} y_{ci}$$

Where *y_ci_* is the binary outcome (1 if the patient is receiving treatment at home, 0 otherwise)

for patient *i* in centre *c*.

*E_c_* is the sum of the predicted probabilities of receiving treatment close to home for all patients in centre *c*:

$$E_{c}=\sum_{i=1}^{n_{c}} \hat{p_{ci}}$$

where $\hat{p_{ci}}$is the predicted probability of receving treatment close to home for patient *i* in centre *c*.

Therefore, the $\text{Log-SIR}_{c}$is given by: Log-SIR

$$\text{Log-SIR}_{c}=\log\left( \frac{O_{c}}{E_{c}} \right)=\log\left( O_{c} \right)-\log\left( E_{c} \right)$$
