# Supplementary material for: Estimating Variance of Log Standardized Incidence Ratios Assessing Health Care Providers’ Performance: Comparative Analysis Using Bayesian, Bootstrap, and Delta Method Approaches
Source: JMIRx Med. 2025 Oct 9;6:e77415. doi: 10.2196/77415 (PMC12605305; doi:10.2196/77415)
Supplement: Multimedia Appendix 2 [file xmed-v6-e77415-s002.docx]

# Appendix B. Delta-method

The delta method is a technique used to approximate the variance of a function of one or more random variables. In the context of estimating the variance of the log standardized incidence ratio Log-SIR, we can apply the delta method to approximate the variance of $\log\left( \frac{O_{c}}{E_{c}} \right)$. It approximates the variance of a function of random variables by using the Jacobian matrix and the covariance matrix of the original variables. For our case, the variance of $\text{Log-SIR}_{c}$ is approximated by:

$\text{Var}\left( \log\text{-SIR}_{c} \right)\approx\nabla g\cdot\text{Cov}\left( O_{c},E_{c} \right)\cdot\nabla g^{\top}$n (B.1)

First, let’s define the covariance matrix of *O_c_* and *E_c_*:

$$\text{Cov(}O_{c},E_{c}=\binom{\text{Var(}O_{c}) Cov(O_{c},E_{c})}{Cov\left( O_{c},E_{c} \right) \text{Var(}E_{c})}$$

The Jacobian matrix (gradient) ∇*g* of the function$g\left( O_{c},E_{c} \right)=\log\left( \text{SMR}_{c} \right)=\log\left( \frac{O_{c}}{E_{c}} \right)$ with respect to *O_c_* and *E_c_* is derived as:

$$\nabla g=\left( \begin{matrix} \frac{\partial}{\partial O_{c}}\log\left( \frac{O_{c}}{E_{c}} \right) & \frac{\partial}{\partial E_{c}}\log\left( \frac{O_{c}}{E_{c}} \right) \end{matrix} \right)$$

And calculating the partial derivatives with respect to the quantities, we get:

$$\frac{\partial}{\partial O_{c}}\log\left( \frac{O_{c}}{E_{c}} \right)=\frac{1}{O_{c}},\quad\frac{\partial}{\partial E_{c}}\log\left( \frac{O_{c}}{E_{c}} \right)=-\frac{1}{E_{c}}$$

Therefore, the Jacobian matrix is:

$$\nabla g=\left( \begin{matrix} \frac{1}{O_{c}} & -\frac{1}{E_{c}} \end{matrix} \right)$$

Substituting ∇*g* and Cov(*O_c_,E_c_*) into formula (9), we get:

$$\text{Var}\left( \text{Log-SIR}_{c} \right)\approx\left( \begin{matrix} \frac{1}{O_{c}} & -\frac{1}{E_{c}} \end{matrix} \right)\binom{\text{Var(}O_{c}) Cov(O_{c},E_{c})}{Cov\left( O_{c},E_{c} \right) \text{Var(}E_{c})}\left( \begin{matrix} \frac{1}{O_{c}}-\frac{1}{E_{c}} \end{matrix} \right)$$

To simplify the derivation, we perform the matrix multiplication step by step:

First, multiply the Jacobian matrix by the covariance matrix:

$$\left( \begin{matrix} \frac{1}{O_{c}} & -\frac{1}{E_{c}} \end{matrix} \right)\binom{\text{Var(}O_{c}) Cov(O_{c},E_{c})}{Cov\left( O_{c},E_{c} \right) \text{Var(}E_{c})}=\left( \frac{\text{Var}\left( O_{c} \right)}{O_{c}}-\frac{\text{Cov}\left( O_{c},E_{c} \right)}{E_{c}},\frac{\text{Cov}\left( O_{c},E_{c} \right)}{O_{c}}-\frac{\text{Var}\left( E_{c} \right)}{E_{c}} \right)$$

Then, multiply the resulting vector by the transpose of the Jacobian matrix:

$$\left( \frac{\text{Var}\left( O_{c} \right)}{O_{c}}-\frac{\text{Cov}\left( O_{c},E_{c} \right)}{E_{c}},\frac{\text{Cov}\left( O_{c},E_{c} \right)}{O_{c}}-\frac{\text{Var}\left( E_{c} \right)}{E_{c}} \right)\cdot\binom{\frac{1}{O_{c}}}{-\frac{1}{E_{c}}}=\left( \frac{\text{Var}\left( O_{c} \right)}{O_{c}}-\frac{\text{Cov}\left( O_{c},E_{c} \right)}{E_{c}} \right)\cdot\frac{1}{O_{c}}$$

$$+\left( \frac{\text{Cov}\left( O_{c},E_{c} \right)}{O_{c}}-\frac{\text{Var}\left( E_{c} \right)}{E_{c}} \right)\cdot\left( -\frac{1}{E_{c}} \right)$$

Finally, we get the $\text{Var}\left( \text{Log-SIR}_{c} \right)$ shown in equation (3)

$$=\frac{\text{Var}\left( O_{c} \right)}{O_{c}^{2}}-\frac{\text{Cov}\left( O_{c},E_{c} \right)}{O_{c}E_{c}}-\frac{\text{Cov}\left( O_{c},E_{c} \right)}{O_{c}E_{c}}+\frac{\text{Var}\left( E_{c} \right)}{E_{c}^{2}}=\frac{\text{Var}\left( O_{c} \right)}{O_{c}^{2}}+\frac{\text{Var}\left( E_{c} \right)}{E_{c}^{2}}-2\frac{\text{Cov}\left( O_{c},E_{c} \right)}{O_{c}E_{c}}$$

In the next, we will derive estimates for the Var(O*_c_*), Var(E*_c_*) and Cov(O*_c_,*E*_c_*).

## Variance of *O_c_*: Var(O*_c_*)

The observed count *O_c_* for a centre *c* is defined as the sum of individual binary outcomes

*Y_i_*:

$$O_{c}=\sum_{i\in n_{c}} Y_{i}$$

Where *Y_i_* is a Bernoulli random variable (taking values 0 or 1), indicating whether an event occurs for individual *i*.

Since *O_c_* is the sum of correlated random variables, its variance expands as:

$\text{Var}\left( O_{c} \right)=\sum_{i\in n_{c}} \text{Var}\left( Y_{i} \right)+2\sum_{i<j\in n_{c}} \text{Cov}\left( Y_{i},Y_{j} \right)$ (B.3)

This follows from the general variance formula for sums of correlated random variables:

$\text{Var}\left( \sum X_{i} \right)=\sum\text{Var}\left( X_{i} \right)+2\sum_{i<j} \text{Cov}\left( X_{i},X_{j} \right)$ (B.4)

*Variance of Y_i_*

For a binary outcome *Y_i_* with probability *p_i_* = *P*(*Y_i_* = 1), the variance is:

$\text{Var}\left( Y_{i} \right)=p_{i}\left( 1-p_{i} \right)$ (B.5)

which is the standard variance formula for a Bernoulli-distributed variable.

*Covariance Between Y_i_ and Y_j_*

To derive the covariance term $\text{Cov}\left( Y_{i},Y_{j} \right)$, we assume a logistic regression model with random intercepts. The probability *p_i_* is modelled as:

$\text{logit}\left( p_{i} \right)=X_{i}\beta+u_{c},$ (B.6)

where *X_i_* are fixed covariates, *β* is the fixed effect coefficient, and *u_c_* is the random effect for centre *c*, assumed to be normally distributed as $u_{c}\sim N\left( 0,\sigma_{u}^{2} \right)$.

Since *Y_i_* and *Y_j_* share the same random effect *u_c_*, they become correlated through *u_c_*.

Using standard results from generalized linear mixed models (GLMMs), the covariance between two binary outcomes *Y_i_* and *Y_j_* in the same group (centre *c*) is approximately:

$\text{Cov}\left( Y_{i},Y_{j} \right)\approx p_{i}\left( 1-p_{i} \right)p_{j}\left( 1-p_{j} \right)\sigma_{u}^{2}$ (B.7)

This approximation comes from the fact that when a common random effect *u_c_* influences both *Y_i_* and *Y_j_*, their correlation is roughly proportional to the variance of the random effect, *σ_u_*^2^. Specifically:

$\text{Corr}\left( Y_{i},Y_{j} \right)\approx\sigma_{u}^{2}$ (B.8)

Multiplying by the standard deviations of *Y_i_* and *Y_j_* , respectively, $\sqrt{p_{i}\left( 1-p_{i} \right)}$, and $\sqrt{p_{j}\left( 1-p_{j} \right)}$, we obtain

$\text{Cov}\left( Y_{i},Y_{j} \right)=\text{Corr}\left( Y_{i},Y_{j} \right)\cdot\text{SD}\left( Y_{i} \right)\cdot\text{SD}\left( Y_{j} \right)$ (B.9)

$\approx\sigma_{u}^{2}\cdot\sqrt{p_{i}\left( 1-p_{i} \right)}\cdot\sqrt{p_{j}\left( 1-p_{j} \right)}$ (B.10)

$=p_{i}\left( 1-p_{i} \right)p_{j}\left( 1-p_{j} \right)\sigma_{u}^{2}$ (B.11)

*Final Expression for Variance of O_c_*

Substituting the variance and covariance terms into the variance expansion formula, we obtain:

$\text{Var}\left( O_{c} \right)=\sum_{i\in n_{c}} p_{i}\left( 1-p_{i} \right)+2\sum_{i<j\in n_{c}} p_{i}\left( 1-p_{i} \right)p_{j}\left( 1-p_{j} \right)\sigma_{u}^{2}$ (B.12)

This accounts for both the independent variation in individual outcomes and the correlation induced by the shared random effect.

## Variance of *E_c_*: Var(E*_c_*)

The expected counts *E* are the sum of predicted probabilities *p_i_* for individuals within a hospital. The variance of *E* arises from the uncertainty in the predicted probabilities due to the random effects.

*Derivation of Var*(*E_c_*)

The expected counts for centre *c* are:

$$E_{c}=\sum_{i\in n_{c}} p_{i}$$

The variance of *E_c_* is:

$$\text{Var}\left( E_{c} \right)=\sum_{i\in n_{c}} \text{Var}\left( p_{i} \right)+2\sum_{i<j\in n_{c}} \text{Cov}\left( p_{i},p_{j} \right)$$

For the random-effects logistic regression model:

$$\text{Var}\left( p_{i} \right)\approx\left[ p_{i}\left( 1-p_{i} \right) \right]^{2}\text{Var}\left( \eta_{i} \right)$$

where $(\eta_{i}=\boldsymbol{x}_{\boldsymbol{i}}^{\boldsymbol{T}}\beta+u_{c})$ is the linear predictor. The covariance between *p_i_* and *p_j_* (for$(i \neq j)$) is:

$$\text{Cov}\left( p_{i},p_{j} \right)\approx\left[ p_{i}\left( 1-p_{i} \right) \right]\left[ p_{j}\left( 1-p_{j} \right) \right]\text{Cov}\left( \eta_{i},\eta_{j} \right)$$

Since $\eta_{i}$ and $\eta_{j}$ share the same random effect $u_{c}$:

$$\text{Cov}\left( \eta_{i},\eta_{j} \right)=\sigma_{u}^{2}$$

Thus:

$$\text{Cov}\left( p_{i},p_{j} \right)\approx\left[ p_{i}\left( 1-p_{i} \right) \right]\left[ p_{j}\left( 1-p_{j} \right) \right]\sigma_{u}^{2}$$

Combining these results:

$\text{Var}\left( E_{c} \right)=\sum_{i\in n_{c}} \left[ p_{i}\left( 1-p_{i} \right) \right]^{2}\text{Var}\left( \eta_{i} \right)+2\sum_{i<j\in n_{c}} \left[ p_{i}\left( 1-p_{i} \right) \right]\left[ p_{j}\left( 1-p_{j} \right) \right]\sigma_{u}^{2}$ (B.13)

## Covariance of *O_c_* and *E_c_*: $\text{Cov}\left( \text{O}_{\text{i}},\text{E}_{\text{i}} \right)$

The covariance between *O* and *E* arises because both depend on the same underlying probabilities *p_i_*, which are influenced by centre level shared random effect.

*Derivation of* $\text{Cov}\left( \text{O}_{\text{i}},\text{E}_{\text{i}} \right)$

To derive the covariance Cov(*O_c_,E_c_*), where *O_c_* is the observed count and *E_c_* is the expected count, we have the definitions:

$O_{c}=\sum_{i\in n_{c}} Y_{i}$and $E_{c} =\sum_{i\in n_{c}} p_{i}$

where *Y_i_* is a binary outcome indicating an event occurrence for individual *i* in centre *c*, and *p_i_* is the corresponding probability.

Using the linearity property of covariance, the covariance between *O_c_* and *E_c_* is:

$$\text{Cov}\left( O_{c},E_{c} \right)=\text{Cov}\left( \sum_{i\in n_{c}} Y_{i},\sum_{i\in n_{c}} p_{i} \right)$$

And if we expand using the property of covariance for sums, we get:

$\text{Cov}\left( O_{c},E_{c} \right)=\sum_{i\in h} \text{Cov}\left( Y_{i},p_{i} \right)+2\sum_{i<j\in h} \text{Cov}\left( Y_{i},p_{j} \right)$

In the following, we derive $\text{Cov}\left( Y_{i},p_{i} \right)$ and $\text{Cov}\left( Y_{i},p_{i} \right)$ for $( i \neq j )$

Since *Y_i_* follows a Bernoulli distribution:

$E\left[ Y_{i} \right]=p_{i},$ and $\text{Var}\left( Y_{i} \right)=p_{i}\left( 1-p_{i} \right)$

The probability *p_i_* depends on the linear predictor *η_i_*, which includes both fixed and random effects:

$\eta_{i}=X_{i}\beta+u_{c}$*,*

where *X_i_* represents the fixed effect covariates and *u_c_* is the hospital-specific random effect.

Since$p_{i}=\text{logit}^{-1}\left( \eta_{i} \right)$, a first-order Taylor series approximation around $E\left[ \eta_{i} \right]$gives:

$$\text{Var}\left( p_{i} \right)\approx p_{i}\left( 1-p_{i} \right)\text{Var}\left( \eta_{i} \right)$$

Thus,

$$\text{Cov}\left( Y_{i},p_{i} \right)=p_{i}\left( 1-p_{i} \right)\text{Var}\left( \eta_{i} \right)$$

$\text{Cov}\left( Y_{i},p_{j} \right)$ *for* $i \neq j$

For individuals *i* and *j* in the same hospital, their probabilities share the same random effect *u_c_*, inducing correlation:

$$\text{Cov}\left( Y_{i},p_{j} \right)\approx p_{i}\left( 1-p_{i} \right)p_{j}\left( 1-p_{j} \right)\sigma_{u}^{2}$$

Substituting these results into our earlier equation, we obtain final expression for the $\text{Cov}\left( O_{c},E_{c} \right)$:

$\text{Cov}\left( O_{c},E_{c} \right)=\sum_{i\in n_{c}} p_{i}\left( 1-p_{i} \right)\text{Var}\left( \eta_{i} \right)+2\sum_{i<j\in n_{c}} p_{i}\left( 1-p_{i} \right)p_{j}\left( 1-p_{j} \right)\sigma_{u}^{2}$ (B.14)
