# Supplementary material for: Estimating Variance of Log Standardized Incidence Ratios Assessing Health Care Providers’ Performance: Comparative Analysis Using Bayesian, Bootstrap, and Delta Method Approaches
Source: JMIRx Med. 2025 Oct 9;6:e77415. doi: 10.2196/77415 (PMC12605305; doi:10.2196/77415)
Supplement: Multimedia Appendix 3 [file xmed-v6-e77415-s003.docx]

Appendix C. Bayesian approach

Model Specification

Consider *y_ci_* as a binary outcome for individual patient *i* in centre (or hospital) *c*. In addition, let *u_c_* be the random effect for centre *c*. The model assumes that the probability of *y_ci_* = 1 is given by a logistic regression:

$$P\left( y_{ci}=1 \mid\boldsymbol{x}_{\boldsymbol{ci}},u_{c},\boldsymbol{\beta} \right)=\text{logit}^{-1}\left( \boldsymbol{x}_{\boldsymbol{ci}}^{\top\boldsymbol{\beta}}+u_{c} \right)=\frac{1}{1+\exp\left( -\left( \boldsymbol{x}_{\boldsymbol{ci}}^{\top\boldsymbol{\beta}}+u_{c} \right) \right)}$$

where **x***_ci_* is a vector of covariates for individual patient *i* in centre *c*, and *β* is a vector of fixed effects.

- The hierarchical model assumes that the random effects *u_c_* are normally distributed:

$$u_{c}\mid\alpha\sim\mathcal{N}\left( \mu_{u},\sigma_{u}^{2} \right)$$

where *µ_u_* and *σ_u_*^2^ are hyperparameters (e.g., *µ_u_* = 0 is common) and hence, the density

function for *u_c_* is the normal distribution given by:

$$f\left( u_{c} \right)=\frac{1}{\sqrt{2\pi\sigma_{u}^{2}}}\exp\left( -\frac{u_{c}^{2}}{2\sigma_{u}^{2}} \right)$$

Likelihood Function

The likelihood for the binary outcome *y_ci_* given the parameters *β* and *u_c_* is:

$$f\left( y_{ci} \mid\boldsymbol{x}_{\boldsymbol{ci}},u_{c},\boldsymbol{\beta} \right)=\left[ \text{logit}^{-1}\left( \boldsymbol{x}_{\boldsymbol{ci}}^{\top\boldsymbol{\beta}}+u_{c} \right) \right]^{y_{ci}}\left[ 1-\text{logit}^{-1}\left( \boldsymbol{x}_{\boldsymbol{ci}}^{\top\boldsymbol{\beta}}+u_{c} \right) \right]^{1-y_{ci}}$$

The likelihood for all observations within centre *c* is:

$$L_{i}\left( \boldsymbol{\beta},u_{c} \right)=\prod_{i=1}^{n_{c}} f\left( y_{ci} \mid\boldsymbol{x}_{\boldsymbol{ci}},u_{c},\boldsymbol{\beta} \right)$$

where *n_c_* is the number of observations in centre *c*.

Prior Distributions

Let’s assume that *β* follows a multivariate normal prior distribution:

$$\pi\left( \boldsymbol{\beta} \right)\sim\mathcal{N}\left( \mu_{\beta},\Sigma_{\beta} \right)$$

This implies that the prior density for *β* is:

$$\pi\left( \boldsymbol{\beta} \right)=\frac{1}{\sqrt{\left( 2\pi\right)^{p}\left| \Sigma_{\beta} \right|}}\exp\left( -\frac{1}{2}\left( \boldsymbol{\beta}-\mu_{\beta} \right)^{\top}\Sigma_{\beta}^{-1}\left( \boldsymbol{\beta}-\mu_{\beta} \right) \right)$$

where, *µ_β_* is the mean vector for *β*, Σ*_β_* is the covariance matrix for *β*, and *p* is the dimension

of *β*.

For the random effects, we have:

$$\pi\left( u_{c} \mid\alpha\right)=\frac{1}{\sqrt{2\pi\sigma_{u}^{2}}}\exp\left( -\frac{\left( u_{c}-\mu_{u} \right)^{2}}{2\sigma_{u}^{2}} \right)$$

Hyperparameters *α* = (*µ_u_,σ_u_*^2^) might also have their own priors, but for simplicity, we’ll focus on *u_c_*.

Posterior Distribution

The posterior distribution for the parameters *β* and *u_c_* given the data **y** and covariates **X** is:

$$\pi\left( \boldsymbol{\beta},u \mid\boldsymbol{y},\boldsymbol{X} \right)\propto L\left( \boldsymbol{\beta},u \mid\boldsymbol{y},\boldsymbol{X} \right)\pi\left( \boldsymbol{\beta} \right)\prod_{i=1}^{I} \pi\left( u_{c} \mid\alpha\right)$$

Substituting the likelihood and the prior distributions:

$$\pi\left( \boldsymbol{\beta},u \mid\boldsymbol{y},\boldsymbol{X} \right)\propto\prod_{c=1}^{C} \left[ \prod_{j=1}^{n_{c}} \left[ \text{logit}^{-1}\left( \boldsymbol{x}_{\boldsymbol{ci}}^{\top\boldsymbol{\beta}}+u_{c} \right) \right]^{y_{ci}}\left[ 1-\text{logit}^{-1}\left( \boldsymbol{x}_{\boldsymbol{ci}}^{\top\boldsymbol{\beta}}+u_{c} \right) \right]^{1-y_{ci}} \right]\times\pi\left( \boldsymbol{\beta} \right)\times\prod_{c=1}^{C} \pi\left( u_{c} \mid\alpha\right)$$

$$\pi\left( \boldsymbol{\beta},u \mid\boldsymbol{y},\boldsymbol{X} \right)\propto\prod_{c=1}^{C} \left[ \prod_{i=1}^{n_{c}} \left[ \frac{e^{\boldsymbol{x}_{\boldsymbol{ci}}^{\top\boldsymbol{\beta}}+u_{c}}}{1+e^{\boldsymbol{x}_{\boldsymbol{ci}}^{\top\boldsymbol{\beta}}+u_{c}}} \right]^{y_{ci}}\left[ \frac{1}{1+e^{\boldsymbol{x}_{\boldsymbol{ci}}^{\top\boldsymbol{\beta}}+u_{c}}} \right]^{1-y_{ci}} \right]\times\pi\left( \boldsymbol{\beta} \right)\times\prod_{c=1}^{C} \pi\left( u_{c} \mid\alpha\right)$$

Final Posterior Distribution with Priors for *β* and *u_c_*

Now, we incorporate the prior *π*(*u_c_* | *α*) into the posterior distribution:

$$\pi\left( \boldsymbol{\beta},u \mid\boldsymbol{y},\boldsymbol{X} \right)\prod_{c=1}^{C} \prod_{i=1}^{n_{c}} \left[ \frac{1}{1+e^{-\left( \boldsymbol{x}_{\boldsymbol{ci}}^{\top\boldsymbol{\beta}}+u_{c} \right)}} \right]^{y_{ci}}\left[ 1-\frac{1}{1+e^{-\left( \boldsymbol{x}_{\boldsymbol{ci}}^{\top\boldsymbol{\beta}}+u_{c} \right)}} \right]^{1-y_{ci}}\times\pi\left( \boldsymbol{\beta} \right)\prod_{c=1}^{C} \frac{1}{\sqrt{2\pi\sigma_{u}^{2}}}\exp\left( -\frac{u_{c}^{2}}{2\sigma_{u}^{2}} \right)$$

This can be written as:

$$\pi\left( \boldsymbol{\beta},u \mid\boldsymbol{y},\boldsymbol{X} \right)\prod_{c=1}^{C} \prod_{i=1}^{n_{c}} \left[ \frac{1}{1+e^{-\left( \boldsymbol{x}_{\boldsymbol{ci}}^{\top\boldsymbol{\beta}}+u_{c} \right)}} \right]^{y_{ci}}\left[ 1-\frac{1}{1+e^{-\left( \boldsymbol{x}_{\boldsymbol{ci}}^{\top\boldsymbol{\beta}}+u_{c} \right)}} \right]^{1-y_{ci}}\times\prod_{c=1}^{C} \frac{1}{\sqrt{2\pi\sigma_{u}^{2}}}\exp\left( -\frac{u_{c}^{2}}{2\sigma_{u}^{2}} \right)\pi\left( \boldsymbol{\beta} \right)$$

The full posterior distribution now becomes:

$$\pi\left( \boldsymbol{\beta},u \mid\boldsymbol{y},\boldsymbol{X} \right)\prod_{c=1}^{C} \prod_{i=1}^{n_{c}} \left[ \frac{1}{1+e^{-\left( \boldsymbol{x}_{\boldsymbol{ci}}^{\top\boldsymbol{\beta}}+u_{c} \right)}} \right]^{y_{ci}}\left[ 1-\frac{1}{1+e^{-\left( \boldsymbol{x}_{\boldsymbol{ci}}^{\top\boldsymbol{\beta}}+u_{c} \right)}} \right]^{1-y_{ci}}\times\prod_{c=1}^{C} \frac{1}{\sqrt{2\pi\sigma_{u}^{2}}}\exp\left( -\frac{u_{c}^{2}}{2\sigma_{u}^{2}} \right)\pi\left( \boldsymbol{\beta} \right)$$

$$\pi\left( \boldsymbol{\beta},u \mid\boldsymbol{y},\boldsymbol{X} \right)\prod_{c=1}^{C} \prod_{i=1}^{n_{c}} \left[ \frac{1}{1+e^{-\left( \boldsymbol{x}_{\boldsymbol{ci}}^{\top\boldsymbol{\beta}}+u_{c} \right)}} \right]^{y_{ci}}\left[ 1-\frac{1}{1+e^{-\left( \boldsymbol{x}_{\boldsymbol{ci}}^{\top\boldsymbol{\beta}}+u_{c} \right)}} \right]^{1-y_{ci}}\times\prod_{c=1}^{C} \frac{1}{\sqrt{2\pi\sigma_{u}^{2}}}\exp\left( -\frac{u_{c}^{2}}{2\sigma_{u}^{2}} \right)\frac{1}{\sqrt{\left( 2\pi\right)^{p}\left| \Sigma_{\beta} \right|}}\exp\left( -\frac{1}{2}\left( \boldsymbol{\beta}-\mu_{\beta} \right)^{\top}\Sigma_{\beta}^{-1}\left( \boldsymbol{\beta}-\mu_{\beta} \right) \right)$$

$$\pi\left( \boldsymbol{\beta},u \mid\boldsymbol{y},\boldsymbol{X} \right)\prod_{c=1}^{C} \prod_{i=1}^{n_{c}} \left[ \frac{1}{1+e^{-\left( \boldsymbol{x}_{\boldsymbol{ci}}^{\top\boldsymbol{\beta}}+u_{c} \right)}} \right]^{y_{ci}}\left[ 1-\frac{1}{1+e^{-\left( \boldsymbol{x}_{\boldsymbol{ci}}^{\top\boldsymbol{\beta}}+u_{c} \right)}} \right]^{1-y_{ci}}\times\frac{1}{\sqrt{\left( 2\pi\right)^{p}\left| \Sigma_{\beta} \right|}}\exp\left( -\frac{1}{2}\left( \boldsymbol{\beta}-\mu_{\beta} \right)^{\top}\Sigma_{\beta}^{-1}\left( \boldsymbol{\beta}-\mu_{\beta} \right) \right)\times\prod_{c=1}^{C} \frac{1}{\sqrt{2\pi\sigma_{u}^{2}}}\exp\left( -\frac{u_{c}^{2}}{2\sigma_{u}^{2}} \right)$$
